# Cost-effectiveness analysis of stand-alone or combined non-invasive imaging tests for the diagnosis of stable coronary artery disease: results from the EVINCI study

**DOI:** 10.1007/s10198-019-01096-5

**Published:** 2019-08-13

**Authors:** Valentina Lorenzoni, Stefania Bellelli, Chiara Caselli, Juhani Knuuti, Stephen Richard Underwood, Danilo Neglia, Giuseppe Turchetti, For the EVINCI Investigators, Mikko Pietila, Maija Mäki, Anna Teresinska, Santiago Aguadé-Bruix, Maria Nazarena Pizzi, Giancarlo Todiere, Alessia Gimelli, Massimo Lombardi, Stefano Puzzuoli, Maurizio Mangione, Paolo Marcheschi, Stephen Schroeder, Tanja Drosch, Rosa Poddighe, Giancarlo Casolo, Constantinos Anagnostopoulos, Francesca Pugliese, Francois Rouzet, Dominique Le Guludec, Francesco Cappelli, Serana Valente, Gian Franco Gensini, Camilla Zawaideh, Selene Capitanio, Gianmario Sambuceti, Fabio Marsico, Pasquale Perrone Filardi, Covadonga Fernández-Golfín, Luis M. Rincón, José L. Zamorano, Frank P. Graner, Stephan Nekolla, Michiel A. de Graaf, Arthur J. H. A. Scholte, Michael Fiechter, Julia Stehli, Oliver Gaemperli, Philipp A. Kaufmann, Eliana Reyes, Sandy Nkomo, Clara Carpeggiani, Daniela Giannessi, Fabio Mariani, Martina Marinelli, Rosa Sicari

**Affiliations:** 1 Institute of Management, Scuola Superiore Sant’Anna, Piazza Martiri della Libertà n. 33, 56127 Pisa, Italy; 2 Institute of Clinical Physiology, CNR, Pisa, Italy; 3 Turku PET Center, University of Turku and Turku University Hospital, Turku, Finland; 4 Biomedical Research Unit, Royal Brompton Hospital and National Heart and Lung Institute, Imperial College London, London, UK; 5 Fondazione CNR Regione Toscana G. Monasterio, Pisa, Italy

**Keywords:** Coronary artery disease, Economic, Cost-effectiveness, Coronary computed tomography, Angiography, Invasive coronary angiography, Stress-imaging, I12, I18

## Abstract

**Aim:**

This study aimed at evaluating the cost-effectiveness of different non-invasive imaging-guided strategies for the diagnosis of obstructive coronary artery disease (CAD) in a European population of patients from the Evaluation of Integrated Cardiac Imaging in Ischemic Heart Disease (EVINCI) study.

**Methods and results:**

Cost-effectiveness analysis was performed in 350 patients (209 males, mean age 59 ± 9 years) with symptoms of suspected stable CAD undergoing computed tomography coronary angiography (CTCA) and at least one cardiac imaging stress-test prior to invasive coronary angiography (ICA) and in whom imaging exams were analysed at dedicated core laboratories. Stand-alone stress-tests or combined non-invasive strategies, when the first exam was uncertain, were compared. The diagnostic end-point was obstructive CAD defined as > 50% stenosis at quantitative ICA in the left main or at least one major coronary vessel. Effectiveness was defined as the percentage of correct diagnosis (cd) and costs were calculated using country-specific reimbursements. Incremental cost-effectiveness ratios (ICERs) were obtained using per-patient data and considering “no-imaging” as reference. The overall prevalence of obstructive CAD was 28%. Strategies combining CTCA followed by stress ECHO, SPECT, PET, or stress CMR followed by CTCA, were all cost-effective. ICERs values indicated cost saving from − 969€/cd for CMR-CTCA to − 1490€/cd for CTCA-PET, − 3092€/cd for CTCA-SPECT and − 3776€/cd for CTCA-ECHO. Similarly when considering early revascularization as effectiveness measure.

**Conclusion:**

In patients with suspected stable CAD and low prevalence of disease, combined non-invasive strategies with CTCA and stress-imaging are cost-effective as gatekeepers to ICA and to select candidates for early revascularization.

**Electronic supplementary material:**

The online version of this article (10.1007/s10198-019-01096-5) contains supplementary material, which is available to authorized users.

## Introduction

Currently, available guidelines for symptomatic patients with suspected stable coronary artery disease (CAD) recommend diagnostic flow-charts using computed tomography coronary angiography (CTCA) as the first-line investigation in all patients [1] or stress imaging and CTCA according to the pre-test probability (PTP) [2]. Additional non-invasive testing is also considered in the case of uncertain results of the first test [1, 2].

Despite this and in view of the wide range of valid available options, the selection of non-invasive imaging in clinical practice may be influenced by factors not included in guidelines [2]. Moreover, direct referral to invasive coronary angiography (ICA) is not uncommon and may result in a relatively high percentage of negative exams thus inducing potentially avoidable costs [3].

While innovations in medical imaging have brought unquestionable benefits improving diagnostic and prognostic accuracy of non-invasive pathways, that success is counterbalanced by an escalation of imaging costs. Evidence suggests that the cost of diagnostic imaging has nearly doubled in the past few decades and there is also a wide variation among countries, due to different practice, healthcare and reimbursement systems [4, 5]; thus the rapid spread of cardiovascular disease in Europe and in fact worldwide, combined with the increasing use of testing via non-invasive approaches, suggest the need for guidance in appropriate decision making.

Accordingly, studies reporting results about cost-effectiveness evaluation of strategies for CAD have slightly increased over the years [6].

In detail, as for clinical studies [7], available economic evaluations of diagnostic strategies for CAD have varied widely regarding the alternatives compared and the methodological approaches used [6]; moreover, the different nature and organization of healthcare systems worldwide and variability of cost over different contexts strongly affect results from economic evaluations. Thus, if clinical research has provided consistent evidence encouraging the use of both CTA and functional tests in the diagnostic pathway [7], the economic perspective is still an open challenge.

In detail, currently available economic studies frequently fail to map all possible options that became available for the diagnosis of stable CAD; many of them use different reference strategies (i.e., primarily ICA and CTCA) and indeed most studies focused on the comparison of a single test failing to produce evidence on a combination of different options; contrary to the direction in which the results of the clinical studies are going. As recent reviews of economic studies highlighted, results from available economic evaluations of non-invasive imaging strategy for stable CAD remains controversial, with no modality consistently emerging as superior to all others and across all patient subgroups [7, 8] and thus not useful to orient clinical practice.

The Evaluation of Integrated Cardiac Imaging for the Detection and Characterization of Ischemic Heart Disease (EVINCI) study (ClinicalTrials.gov Identifier: NCT00979199) was a multicenter, multinational European, non-randomized controlled clinical trial intended to compare the performance of CTCA and available non-invasive stress-imaging modalities for the diagnosis of obstructive CAD in patients with stable chest pain symptoms, intermediate PTP and without prior evidence of disease. The main results of the EVINCI study have been reported elsewhere [9].

The health economic analysis was a major secondary aim of the study, and the EVINCI design allowed comparing strategies involving single modalities or combinations of CTCA and stress imaging for diagnostic cost-effectiveness with the potential of providing valuable evidence and resolving some limitations of available evidence, first and foremost with respect to the evaluation of strategies combining imaging modalities.

## Materials and methods

### Study population and data

Per-patient data from the EVINCI study were used in the present analysis. Briefly, the EVINCI trial prospectively enrolled patients with symptoms of suspected stable CAD, without prior evidence of disease and intermediate PTP, after exclusion of those subjects reclassified to very low or high risk after performing an exercise ECG [9]. The study was performed in 13 centers from eight different European countries. By protocol, patients underwent CTCA and at least one imaging stress-test from among cardiac magnetic resonance (CMR), echocardiography (ECHO) positron emission tomography (PET) or single photon computed emission tomography (SPECT). All patients with at least one positive non-invasive exam were referred to ICA. Standard acquisition and analysis protocols were agreed on for each technique, image quality analysis and reporting was performed independently at each recruiting center and at dedicated core-laboratories (where observers were blinded to the clinical data and to any other test results). Among the 697 patients initially enrolled, 475 patients completed the entire protocol. To ensure homogeneous evaluation of imaging tests, only data from the 350 patients who had complete core-laboratory evaluation were used in the present analysis. Analyses were repeated in the subgroup of patients (*N* = 101) having complete data on ICA, CTCA, and both ECHO and SPECT, the most frequently used imaging stress tests.

### Diagnostic strategies

**Fig. 1:**
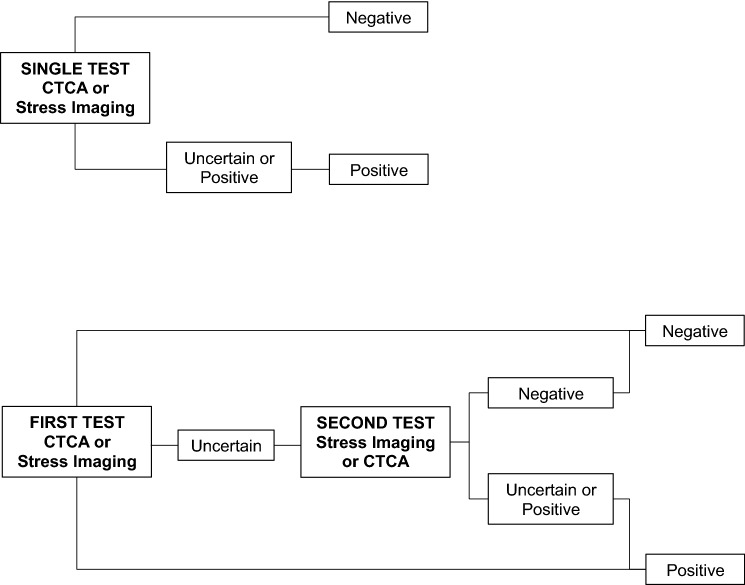
Each single or combined imaging strategy was defined as positive or negative as schematically shown

Thirteen strategies involving CTCA and single imaging stress-tests or their combinations were evaluated. Positivity criteria and quality evaluation at core-lab analysis for each non-invasive test were detailed in Neglia et al. [9]. Any test was defined as uncertain by core-labs if the image quality was judged as suboptimal. Stress tests were also defined as uncertain if they did not show abnormalities but the stress protocols were inadequate (submaximal stress, therapy not withdrawn). According to Guidelines [2], combined strategies were built with CTCA as the first test followed by one stress-imaging in case of uncertain CTCA results or with one stress-imaging as first test followed by CTCA in case of uncertain stress-test results. For the cost-effectiveness analysis each strategy was defined as positive or negative as schematically shown in Fig. 1.

Obstructive CAD was defined as > 50% diameter stenosis in the left main or at least one major coronary vessel by quantitative ICA at core-lab analysis. For all imaging-guided diagnostic strategies true positives (TP) or true negatives (TN) (correct diagnoses) were defined in concordance with ICA results. In patients who did not perform invasive evaluation due to all negative non-invasive exams, and in whom negativity was confirmed by core-labs, all strategies were assumed to be true negatives. For the cost-effectiveness analysis, a “no-imaging” strategy was taken as reference and direct referral to ICA was considered as a comparative pathway. The “no-imaging” strategy was assumed to provide a correct diagnosis only in patients with negative ICA, while the direct invasive strategy was assumed to provide a correct diagnosis in all patients.

### Definition of effectiveness and costs

Effectiveness was defined in terms of correct diagnosis. For each patient and for each strategy, a dichotomous variable of effectiveness was defined taking the value of “one” in case of concordance with ICA results and the value of “zero” in case of discordance.

Country-specific charge collected through the EVINCI study was used to quantify costs associated with single tests and combinations. In particular, in each participating center, clinicians and administrative personnel filled a dedicated case report form including code, description, charge and reimbursement regimen for different tests. Collected data were checked against national administrative data, when available, and inconsistencies were solved by direct contacts with the center. Overall costs of the different strategies were thus estimated for each patient adding the specific cost data to costs associated with false negative or false positive results. In published reports [10–13], the cost of a false negative imaging test has been variably estimated as being 2–6 times the cost of ICA, on the assumption that it could lead to additional testing later on. In the present study, using a conservative approach, the cost of a false negative strategy was assumed to be twice the cost of ICA. The cost of a false positive strategy was assumed to be the cost of an unnecessary ICA. In the case of the “no-imaging” strategy the cost was only related with false negatives. All costs were converted to Euro 2012 using the official exchange rate [14] and then adjusted by the purchasing power parity (PPP) [15] to account for differences in the opportunity cost of resources across the economies considered [16].

### Cost-effectiveness analysis

The cost-effectiveness analysis was performed using the payer perspective and the diagnostic performance as measure of effectiveness. Using the “no-imaging” strategy as reference, the cost-effectiveness ratio (ICER) was defined as incremental cost for one more correct diagnosis (€/cd) over 100 patients. Mean costs, effectiveness and the cost-effectiveness ratios (CERs) were derived for all the strategies. Strategies were then ranked based on ascendant order of costs and effectiveness. The ICERs were thus derived as the ratio of incremental costs by incremental unit of effectiveness after excluding strategies “dominated” (less effective and more costly than one or more strategies) or subjected to “extended dominance” (dominated by a linear combination of other strategies). The 95% confidence intervals for ICER were computed by means of non-parametric bootstrap with 10,000 replicates and accounting for the hierarchical structure of the data, with patients clustered into countries.

### Scenario analysis

In a scenario analysis, concordance of positive/negative non-invasive strategies results with performed/not-performed early revascularization procedures (within 90 days from enrolment based on independent clinical decision of the local attending physicians) was used as an alternative measure of effectiveness.

### Sub-group analysis

Both the main analysis and the scenario analysis were repeated in the subgroup of the EVINCI population in which each patient had undergone CTCA, stress ECHO, stress SPECT and ICA.

The main cost-effectiveness analysis was also performed separately in the different countries.

## Results

Details of patients’ characteristics, diagnostic performance of single tests and combined non-invasive strategies as well as costs are available as Supplementary data (Tables S1–S3). Briefly, demographics and clinical characteristics of patients undergoing each different imaging were fairly well matched, with patients undergoing PET being less likely males with lower prevalence of family history of CAD and less typical symptoms. The overall prevalence of obstructive CAD was 28% and was similar among groups (Table S1).

The diagnostic performance, as expressed by the percentage of correct diagnoses (TP + TN), was in general higher for combined CTCA and stress imaging strategies than for single non-invasive tests (Table S2).

Country-specific reimbursement for the different non-invasive tests and ICA largely varied across countries and variability remained substantial also when adjusting for PPP (Table S3).

### Cost-effectiveness analysis

Results of the main cost-effectiveness analysis for the diagnosis of obstructive CAD are summarized in Table 1. Mean costs and effectiveness over 100 patients are reported for each strategy together with delta costs, delta effectiveness and ICERs obtained via bootstrap replicates using “no-imaging” as reference. Strategies using stand-alone CTCA, a specific stress-test, their combinations (CTCA first or stress-test first) or direct referral to ICA are compared. Combined non-invasive strategies including CMR-CTCA, CTCA-ECHO, CTCA-PET, CTCA-SPECT and direct referral to ICA were all cost-effective and dominated stand-alone stress-imaging or the other combinations. Stand-alone CTCA had lower induced costs, but only a marginal improvement in effectiveness as compared with “no-imaging”. Cost savings per one additional correct diagnosis over 100 patients ranged from 969 €/cd for CMR-CTCA to 1490 €/cd for CTCA-PET, 3092 €/cd for CTCA-SPECT and 3776€/cd for CTCA-ECHO. Direct referral to ICA was cost-effective but with additional costs.

**Table 1:** Results of the cost-effectiveness analysis of single or combined imaging strategies for the diagnosis of obstructive CAD

	Cost, €	Effectiveness, %	Δ Cost	Δ Effectiveness	ICER (95% CI)
CMR					
No-imaging	98,991	72	–	–	–
CTCA	51,205	73	− 47,811	0.6	− 79,685 (− 153,074; 144,834)
CMR	84,388	80	− 14,763	8.5	Extended dominated
CTCA-CMR	87,203	77	− 11,985	4.9	Dominated
CMR-CTCA	87,209	84	− 11,819	12.2	− 969 (− 2282; 7001)
ICA	165,111	100	66,151	28	2362 (1495; 3504)
ECHO					
No-imaging	98,991	72	–	–	–
ECHO	42,612	55	− 56,414	− 17	Extended dominated
CTCA	51,205	73	− 47,811	0.6	Extended dominated
CTCA-ECHO	51,216	85	− 47,886	12.7	− 3776 (− 6177; − 2740)
ECHO-CTCA	58,781	80	− 40,407	8	Dominated
ICA	165,111	100	66,151	28	2362 (1495; 3504)
PET					
No-imaging	98,991	72	–	–	–
CTCA	51,205	73	− 47,811	0.6	− 79,685 (− 153,074; 144,834)
CTCA-PET	79,901	85	− 19,073	12.8	− 1490 (− 3185; 1393)
PET	117,722	71	18,663	− 0.8	Dominated
PET-CTCA	134,117	76	35,232	4.2	Dominated
ICA	165,111	100	66,151	28	2362 (1495; 3504)
SPECT					
No-imaging	98,991	72	–	–	–
CTCA	51,205	73	− 47,811	0.6	− 79,685 (− 153,074; 144,834)
CTCA-SPECT	74,635	80	− 24,425	7.9	− 3092 (− 7998; − 504)
SPECT	90,125	68	− 9035	− 4.2	Dominated
SPECT-CTCA	103,446	77	4260	4.8	Dominated
ICA	165,111	100	66,151	28	2362 (1495; 3504)

Mean costs and effectiveness over 100 patients are reported together with delta costs, delta effectiveness and ICERs obtained via bootstrap replicates using “no imaging” strategy as reference. Strategies involving CTCA, each stress imaging modality and combinations or direct referral to ICA are compared and listed in order of increasing costs

*cd* correct diagnosis, *CMR* cardiac magnetic resonance, *CTCA* computed-tomography-coronary-angiography, *ECHO* stress-echocardiography, *ICA* invasive-coronary-angiography, *ICER* incremental-cost-effectiveness-ratio, *PET* positron-emission-tomography, *SPECT* single-photon-emission-computed-tomography

**Fig. 2:**
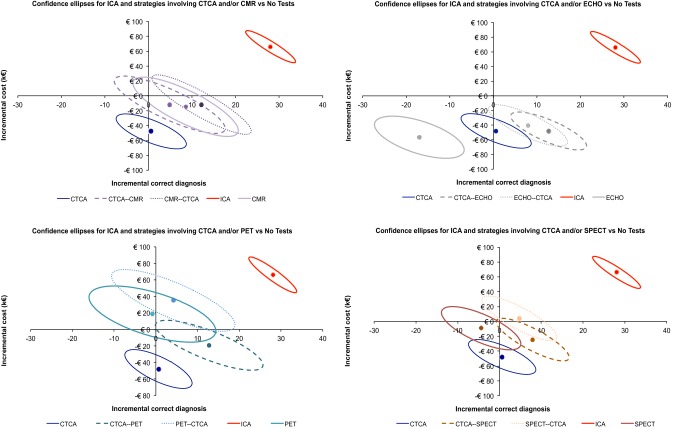
Differences in mean cost and in mean effectiveness (with relative contour plots representing confidence intervals obtained from bootstrap analysis) are plotted in four different cost-effectiveness planes allowing comparison of self-standing CTCA, one stress-imaging and their combinations

The differences in mean cost and in mean effectiveness (with relative contour plots representing confidence intervals of the distribution generated from bootstrap analysis) are shown in Fig. 2. The distribution plot for ICA is located in the upper right quadrant, thus indicating higher effectiveness at the price of higher costs. The distribution plot for stress ECHO is located in the lower left quadrant demonstrating not only reduction of costs but also lower diagnostic effectiveness, while uncertainty exists for all other self-standing tests. For combined strategies, the distribution plots for CMR-CTCA, CTCA-ECHO, CTCA-PET and CTCA-SPECT are consistently located in the lower right quadrants, indicating gain in effectiveness with reduced costs, with some uncertainty in particular for CMR-CTCA.

When considering early revascularization as an alternative measure of effectiveness, combined strategies with CTCA as gatekeeper followed by either stress CMR, ECHO, PET or SPECT were all cost-effective as compared with “no-imaging” and dominated stand-alone or other combined strategies, despite some uncertainty around the point estimate of the ICERs. Direct referral to ICA was cost-effective requiring additional costs (Table 2).

**Table 2:** Results of the cost-effectiveness analysis of single or combined imaging strategies when concordance of imaging results with early revascularization performed was taken as measure of effectiveness

	Cost, €	Effectiveness, %	Δ Cost	Δ Effectiveness	ICER (95% CI)
CMR					
No-imaging	86,694	76	–	–	–
CTCA-CMR	76,200	82	− 10,735	5.9	− 1820 (− 13,138; 10,446)
CTCA	84,369	69	− 2336	− 6.8	Dominated
CMR-CTCA	93,814	81	7135	4.5	Dominated
CMR	96,873	77	9828	0.8	Dominated
ICA	183,268	90	96,518	14.0	6876 (4017; 13,483)
ECHO					
No-imaging	86,694	76	–	–	–
CTCA-ECHO	70,431	78	− 16,300	1.8	− 9312 (− 43,091; 40,926)
ECHO-CTCA	79,122	74	− 7692	− 2.4	Dominated
CTCA	84,369	69	− 2336	− 6.8	Dominated
ECHO	101,899	50	15,073	− 26.2	Dominated
ICA	183,268	90	96,518	14.0	6876 (4017; 13,483)
PET					
No-imaging	86,694	76	–	–	–
CTCA	84,369	69	− 2336	− 6.8	Dominated
CTCA-PET	87,652	80	754	4.3	177 (− 17,104; 16,468)
PET	128,289	67	41,645	− 9.7	Dominated
PET-CTCA	130,037	75	43,507	− 1.8	Dominated
ICA	183,268	90	96,518	14.0	6876 (4017; 13,483)
SPECT					
No-imaging	86,694	76	–	–	–
CTCA-SPECT	83,889	79	− 2683	2.3	1167 (− 21,796; 18,493)
CTCA	84,369	69	− 2336	− 6.8	Dominated
SPECT-CTCA	109,236	74	22,407	− 2.2	Dominated
SPECT	112,447	66	25,491	− 9.4	Dominated
ICA	183,268	90	96,518	14.0	6876 (4017; 13,483)

Mean costs and effectiveness over 100 patients are reported together with delta costs, delta effectiveness and ICERs obtained via bootstrap replicates using “no imaging” strategy as reference. Strategies involving CTCA, each stress imaging modality and combinations or direct referral to ICA are compared and listed in order of increasing costs

*cd* correct diagnosis, *CMR* cardiac magnetic resonance, *CTCA* computed-tomography-coronary-angiography, *ECHO* stress-echocardiography, *ICA* invasive-coronary-angiography, *ICER* incremental-cost-effectiveness-ratio, *PET* positron-emission-tomography, *SPECT* single-photon-emission-computed-tomography

### Subgroup analysis

The main results of the cost-effectiveness analysis were confirmed in the subgroup of 101 patients submitted to CTCA, stress ECHO, stress SPECT and ICA (Table 3). Combined CTCA-ECHO and CTCA-SPECT strategies were cost-effective for the diagnosis of obstructive CAD dominating stand-alone stress-tests and other combinations. Stand-alone CTCA was also cost-effective but provided slightly lower diagnostic accuracy than combined strategies. Direct referral to ICA was cost-effective with higher costs per correct diagnosis. Considering early revascularization as alternative measure of effectiveness, only CTCA-SPECT [ICER − 9520€/cd (95% CI: − 26,872 to − 18,200)] was a cost-effective non-invasive strategy while direct referral to ICA [ICER: 7064€/cd (95% CI: 2214–57,913)] was cost-effective but required additional costs (Table 4).

**Table 3:** Results of the cost-effectiveness analysis of different single or combined imaging strategies for (a) the diagnosis of obstructive CAD performed and (b) early revascularization in a subgroup of 101 patients

	Cost, €	Correct diagnosis	Δ Cost, €	Δ Effectiveness, cd	ICER (95% CI), €/cd
(a)					
No-imaging	120,649	68	–	–	–
CTCA	29,157	82	− 91,273	13.9	− 6566 (− 30,492; − 4284)
CTCA-ECHO	37,462	84	− 83,296	15.9	− 5239 (− 12,176; − 3662)
CTCA-SPECT	46,113	86	− 74,552	17.8	− 4188 (− 7195; − 3068)
ECHO	47,824	48	− 72,940	− 20.8	Dominated
ECHO-CTCA	62,699	78	− 57,989	9.8	Dominated
SPECT	85,583	61	− 35,087	− 6.8	Dominated
SPECT-CTCA	90,803	76	− 29,947	8	Dominated
ICA	159,946	100	39,373	31.6	1246 (87; 3223)
(b)					
No-imaging	95,408	76	–	–	–
CTCA	41,756	73	− 53,724	− 3.1	Extended dominated
ECHO	48,021	40	− 47,176	− 35.9	Dominated
CTCA-ECHO	50,485	75	− 45,047	− 1	Extended dominated
CTCA-SPECT	65,082	79	− 30,351	3.2	− 9520 (− 26,872; 18,200)
ECHO-CTCA	77,107	68	− 18,546	− 7.3	Dominated
SPECT	86,313	59	− 9270	− 16.8	Dominated
SPECT-CTCA	91,506	73	− 3692	− 3.2	Dominated
ICA	184,442	88	89,013	13.6	7064 (2214; 57,913)

Mean costs and effectiveness over 100 patients are reported together with delta costs, delta effectiveness and ICERs obtained via bootstrap replicates using “no imaging” strategy as reference. Strategies involving CTCA, each stress imaging modality and combinations or direct referral to ICA are compared and listed in order of increasing costs

*cd* correct diagnosis, *CTCA* computed-tomography-coronary-angiography, *ECHO* stress-echocardiography, *ICA* invasive-coronary-angiography, *ICER* incremental-cost-effectiveness-ratio, *SPECT* single-photon-emission-computed-tomography

**Table 4:** Results of the effectiveness analysis of different single or combined imaging strategies for early revascularization in a subgroup of 101 patients

	Cost, €	Correct prediction	Δ Cost, €	Δ Effectiveness, cd	ICER (95% CI), €/cd
No-imaging	95,408	76	–	–	–
CTCA	41,756	73	− 53,724	− 3.1	Extended dominated
ECHO	48,021	40	− 47,176	− 35.9	Dominated
CTCA-ECHO	50,485	75	− 45,047	− 1	Extended dominated
CTCA-SPECT	65,082	79	− 30,351	3.2	− 9520 (− 26,872; 18,200)
ECHO-CTCA	77,107	68	− 18,546	− 7.3	Dominated
SPECT	86,313	59	− 9270	− 16.8	Dominated
SPECT-CTCA	91,506	73	− 3692	− 3.2	Dominated
ICA	184,442	88	89,013	13.6	7064 (2214; 57,913)

Mean costs and effectiveness over 100 patients are reported together with delta costs, delta effectiveness and ICERs obtained via bootstrap replicates using “no imaging” strategy as reference. Strategies involving CTCA, each stress imaging modality and combinations or direct referral to ICA are compared and listed in order of increasing costs

*cd* correct diagnosis, *CTCA* computed-tomography-coronary-angiography, *ECHO* stress-echocardiography, *ICA* invasive-coronary-angiography, *ICER* incremental-cost-effectiveness-ratio, *SPECT* single-photon-emission-computed-tomography

**Fig. 3:**
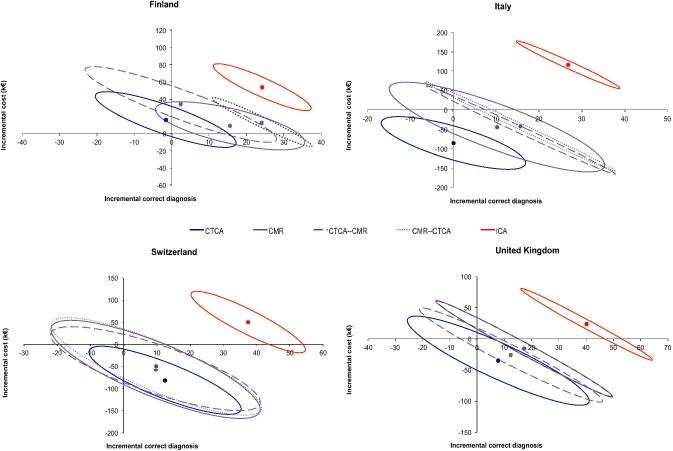
Differences in mean cost and in mean effectiveness (with relative contour plots representing confidence intervals obtained from bootstrap analysis) are plotted in cost-effectiveness planes allowing comparison of self-standing CTCA, CMR and their combinations in different countries

**Fig. 4:**
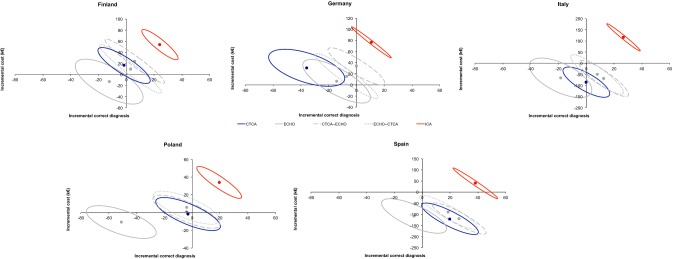
Differences in mean cost and in mean effectiveness (with relative contour plots representing confidence intervals obtained from bootstrap analysis) are plotted in cost-effectiveness planes allowing comparison of self-standing CTCA, ECHO and their combinations in different countries

**Fig. 5:**
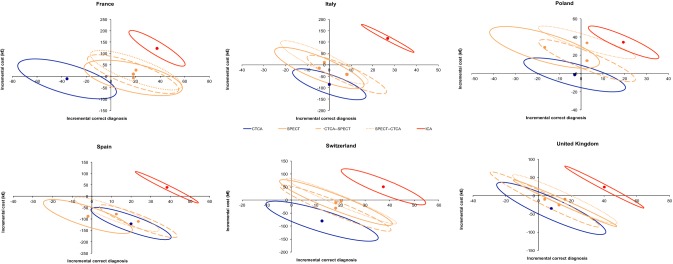
Differences in mean cost and in mean effectiveness (with relative contour plots representing confidence intervals obtained from bootstrap analysis) are plotted in cost-effectiveness planes allowing comparison of self-standing CTCA, SPECT and their combinations in different countries

Results from the country-specific cost-effectiveness analysis indicated high variability among countries and high uncertainty for most of the strategies, mainly due to the limited sample size (Figs. 3, 4 and 5). However, combined strategies improved effectiveness of stand-alone stress-tests or CTCA with few exceptions, while the impact on costs was variable depending on the rate of false test results and on the local costs of the various tests.

## Discussion

**Fig. 6:**
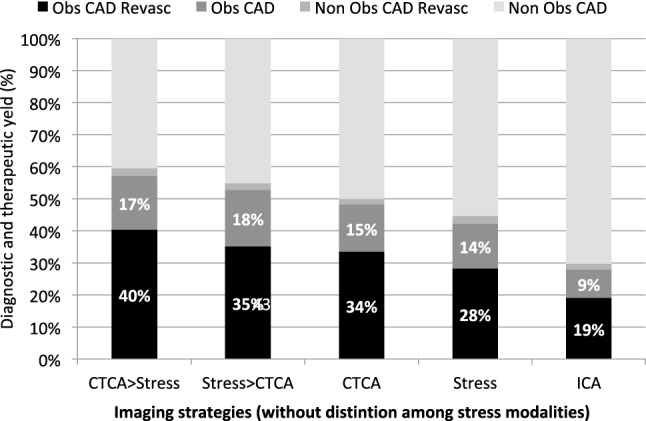
The diagnostic and therapeutic yield of invasive angiography, if indicated on the basis of non-invasive imaging strategies (without distinction among stress modalities) involving CTCA, Stress tests or combination of in different order (CTCA > stress and stress > CTCA). For comparison, the same figures are obtained when all patients would are referred directly to ICA

The economic evaluation of the EVINCI study indicates that non-invasive strategies combining CTCA with stress-imaging, in the case of inconclusive results of the first test, are cost-effective diagnostic options in patients with stable chest pain symptoms and low prevalence of obstructive CAD. CTCA as first test followed by stress ECHO, SPECT, PET or stress CMR as first test followed by CTCA were associated with overall reduced costs and higher diagnostic efficacy, as compared with a “no-imaging” strategy, self-standing single non-invasive tests or other combinations. Direct referral to ICA was also a cost-effective option but required additional costs. In general, whether the combination of CTCA with stress-imaging would be used as gate-keeper to catheterization, the positive yield of ICA for the diagnosis of obstructive CAD would increase from 28 to 47% and the rate of revascularizations performed from 19% up to 40% (Fig. 6).

### What is new for self-standing vs combined non-invasive imaging diagnostic strategies

In detail, the present study highlights the lower performance of self-standing non-invasive imaging modalities as compared with combined strategies and that could be explained by multiple factors. The EVINCI population, as most recent cohorts enrolled in imaging trials [17, 18], had a low prevalence of obstructive CAD (28%). In this low-risk populations, it is expected that the prevalence of extensive disease, able to induce severe ischemia and contractile impairment, is even lower. Thus strategies using self-standing wall motion-based imaging, in particular stress ECHO, are expected to reduce overall costs by reducing the number of ICA exams but will be associated with more false negatives, i.e., unrecognized obstructive CAD. Conversely, perfusion-based stress imaging strategies are expected to have more false positive results driving the additional costs of more unusual ICA. As a matter of fact, in the EVINCI study false positives ranged from 0 to 2.1% for stress CMR and ECHO, to 6.4–13.6% for stress SPECT and PET and false negatives from 13.4–8.5% to 9.1–3.4%, respectively (Table 2). CTCA had fewer false positives than SPECT and PET and fewer false negatives than CMR and ECHO. Moreover, in these “real world” populations, each modality is frequently not exploited at its full potential and a substantial percentage of imaging tests may be inconclusive, as demonstrated by the core-lab analysis of the EVINCI data. Thus, combining CTCA with stress-imaging may reduce uncertain results and improve the diagnostic performance of the combined strategies, thus ultimately reducing the overall costs per correct diagnosis.

The present findings cannot be extended to populations with higher prevalence of disease and do not provide information on the impact on long-term clinical outcome. However, from a health economic perspective they support the diagnostic use of integrated non-invasive imaging for the screening of patients with suspected stable CAD recommended in the 2013 ESC Guidelines [2] and the role of CTCA was recently underlined by the UK National Institute for Health and Clinical Excellence [1].

### Findings from the EVINCI cost-effectiveness analysis and available evidence

Variability in the study design, imaging methods and measures of effectiveness and costs prevents an easy comparison of available studies with the present findings. Many existing studies were performed in selected populations recruited in single or a few expert centers in one nation and did not include all available modalities and combinations. In the CE-MARC study, for example, multi-parametric CMR performed in a single expert center was compared with conventional stress SPECT showing the superiority of strategies including CMR at cost-effectiveness analysis [19] as also documented in other nation-wide studies [20]. The CE-MARC two-trial, performed in a larger population and in multiple centers, could not confirm significant differences between CMR and SPECT [21]. Similarly, in a large prospective study there were no significant differences among stress CMR, ECHO or SPECT strategies in terms of effectiveness or costs [22]. When CTCA and stress-imaging were compared [23, 24] over long-term follow-up, cost differences were minimal without significant differences in patient outcome. Other recent cost-effectiveness analyses based on simulation models using available published data and limiting the diagnostic evaluation to single tests followed by ICA for equivocal or abnormal results, suggested that direct referral to ICA for patients with low/intermediate probability of CAD was not cost-effective [25]. In another recent study where diagnostic outcomes were modelled including imaging-guided treatments, lifetime prognosis was derived using a state-transition model, the results suggested that for patients with stable chest pain and a low/intermediate probability of CAD, CTCA as a triage test followed by cardiac stress imaging was cost effective [26].

Pragmatic randomized controlled trials (RCTs) are beneficial in that they allow follow-up testing and care patterns to occur based on the patient’s overseeing physician and not constrained by trial-directed follow-up care, thereby reflecting current real-world practices. However, they are also limited by possible inconsistency of patients’ management with imaging results and by variability in the outcome measurements. The PROMISE study was the largest recent pragmatic randomized controlled trial randomizing symptomatic patients without known CAD to CTCA or functional testing including stress SPECT, ECHO or ECG [17]. The overall prevalence of obstructive CAD requiring revascularization was even lower than in the EVINCI study (< 5%). Within 90 days of randomization, more patients in the CTCA arm underwent ICA, which showed more frequently obstructive CAD leading to more frequent revascularizations than in the functional testing arm. These differences, however, did not translate into superior outcomes at 25-month follow-up either for the primary composite end-point or secondary end-points. Quality of life and symptoms were similarly improved by both strategies and the economic analysis showed that CTCA and functional diagnostic testing strategies had similar costs through 3 years of follow-up [23, 27], but a lifetime cost-effectiveness analysis was not performed because the hypothesized primary clinical benefits for CTCA were not found.

In the SCOT-HEART, patients were randomized to standard stress testing, including stress SPECT in a minority, or standard stress testing with a CTCA-guided strategy [18]. There was no difference in the number of downstream invasive exams but, in the CTCA arm, ICA was more likely to show obstructive CAD and there was a trend for more early coronary revascularization procedures (< 90 days) with borderline significant reduction in the coronary event rates. In further analyses, the cumulative mean costs of treatment over 6 months was higher in the CCTA group than for standard care alone, mainly attributable to the direct costs of CTCA itself, but a cost-effectiveness analysis was not performed [24].

In summary, in patients with suspected stable CAD and low-to-intermediate prevalence of obstructive disease, one modality or strategy did not consistently emerge as superior to all others from a health economics perspective [7, 28].

Despite the differences with other cost-effectiveness studies, the EVINCI analysis confirmed that no self-standing non-invasive imaging modality is superior to the others but also showed how strategies combining CTCA with stress-imaging can be cost-effectively used to diagnose obstructive CAD and identify candidates to revascularization prior to ICA.

### Improvement and strength of the EVINCI study over the state of the art

The present study improved the state of the art regarding the cost-effectiveness of strategies for the diagnosis of CAD, first of all by providing evidence in a contemporary European population with acceptable geographical and socio-demographical “representativeness”, both updating evidence about self-standing non-invasive diagnostic strategies and also bringing data for the evaluation of combined strategies.

Finally, some other strengths of the EVINCI economic analysis are related to the process of gathering imaging data. These were prospectively obtained in a homogeneous European population of patients with symptoms of suspected stable CAD and low prevalence of disease, and head-to-head comparisons of multiple diagnostic strategies, including combinations, were allowed by the study protocol. Diagnostic effectiveness of tests was also objectively evaluated by independent core-labs and costs assessed in each center.

### Limitations

One limitation of the present study regards the diverse utilization of imaging modalities among the single countries and centers. Those differences, despite reflecting real-practice and the different spread of technologies among the involved centers, limited the direct comparison of all the strategies and of modalities exploited at their full potential. Similarly, this is so for the diverse number of patients assigned to the different strategies in different centers and countries. There were insufficient numbers to perform stratified analyses to account for these differences.

As the EVINCI study was designed for a diagnostic end-point, correct diagnosis of obstructive CAD was used as measure of effectiveness. This implied the assumption of perfect accuracy of ICA, thus potentially giving CTCA an advantage over stress-imaging. On the other hand, in the scenario analysis based on the revascularizations performed, functional imaging was potentially advantaged.

Cost data collected throughout the EVINCI study showed high variability of charges for the different tests among the countries involved (Table S1). Despite this variability, ICA was always the most expensive procedure, while ECHO was the least expensive one, followed by CTCA. SPECT and CMR were generally more expensive than CTCA and associated charges showed the highest variability, even when adjusted for PPP. As previously reported [8, 28, 29] such variability, beyond modelling approaches and assumptions, may have significantly impacted on results. As an additional limitation, the health economic evaluation was performed using charges and these often do not reflect real costs. To address some of these limitations, where possible, analyses were performed considering recommendations for the analysis of multinational trials [30–32], thus using proper methods to adjust costs and to account for the hierarchical structure of the data and also providing country-specific results to overcome problems related to the transferability of results from the overall analysis. Further research is needed to define the possible determinants of this heterogeneity. Nevertheless, the present data may offer relevant information to health-economists for conducting future studies as well as for interpreting the existing ones, in a multi-center and multi-national context.

## Conclusions

In a contemporary European population of patients with suspected stable CAD and low prevalence of disease, diagnostic strategies combining CTCA and stress-imaging are cost-effective as gatekeepers to ICA and for selecting candidates for revascularization. In a scenario of uncertainty about health economic consequences of multiple diagnostic pathways, a rational combination of non-invasive imaging modalities may offer the most cost- and clinically effective t!esting for the single patient.

The present results suggest the clinical and economic relevance of improving the diagnostic performance of imaging tests and potentially limiting inconclusive imaging findings using more updated technologies, exploiting each tes!t at its full potential (i.e., combining perfusion and wall motion or anatomical and functional information) and strictly following correct protocols. They also support the existing recommendations to clearly state when and why an imaging exam is inconclusive in the clinical reports [33] to indicate additional testing before referring the patient to invasive procedures.

Which imaging strategy, beyond its diagnostic performance, is able to achieve the most important goal of effectively guiding treatment to improve outcome in patients with stable CAD is still undetermined. Further large fully comparative multicenter, multinational, multimodality outcome-based imaging trials are needed to provide useful global and country-specific health economic information.

## Electronic supplementary material

Below is the link to the electronic supplementary material.

Supplementary material 1 Supplementary data detail patients’ characteristics (Table S1), diagnostic performance of single tests and combined non-invasive strategies (Table S2), and centers’ specific charges for the diverse tests (Table S3) (DOCX 74 kb)
