# Supplementary material for: Cost-effectiveness analysis of stand-alone or combined non-invasive imaging tests for the diagnosis of stable coronary artery disease: results from the EVINCI study
Source: Eur J Health Econ. 2019 Aug 13;20(9):1437–49. doi: 10.1007/s10198-019-01096-5 (PMC6856023; doi:10.1007/s10198-019-01096-5)
Supplement: Supplementary file 1 — Supplementary material 1 Supplementary data detail patients’ characteristics (Table S1), diagnostic performance of single tests and combined non-invasive strategies (Table S2), and centers’ specific charges for the diverse tests (Table S3) (DOCX 74 kb) [file 10198_2019_1096_MOESM1_ESM.docx]

**Table S1. Baseline demographics and clinical characteristics of patients submitted to different imaging modalities.**

|  | | **CTCA**  **(n=350)** | **CMR**  **(n=82)** | **ECHO**  **(n=189)** | **PET**  **(n=59)** | **SPECT**  **(n=219)** |
| --- | --- | --- | --- | --- | --- | --- |
| **Demographics** |  |  |  |  |  |  |
| Age (years) | | 59±9 | 60±8 | 60±9 | 61±8 | 60±9 |
| Males **(#)** | | 209 (59.7%) | 45 (54.9%) | 104 (55%) | 25 (42.4%) | 146 (66.7%) |
| **Cardiovascular risk factors** |  |  |  |  |  |  |
| Family history of CAD**^#^** | | 115 (32.9%) | 36 (43.9%) | 43 (22.8%) | 13 (22.0%) | 72 (32.9%) |
| Diabetes | | 78 (22.3%) | 22 (26.8%) | 37 (19.6%) | 8 (13.6%) | 61 (27.9%) |
| Hypertension | | 208 (59.4%) | 46 (56.1%) | 119 (63.0%) | 26 (44.1%) | 135 (61.6%) |
| Hypercholesterolemia | | 194 (55.4%) | 50 (61.0%) | 108 (57.1%) | 36 (61.0%) | 129 (58.9%) |
| Obesity | | 73 (20.9%) | 16 (19.5%) | 35 (18.5%) | 8 (13.6%) | 48 (21.9%) |
| Smoking | | 88 (25.1%) | 21 (25.6%) | 45 (23.8%) | 8 (13.6%) | 57 (26.0%) |
| **Symptoms^#^** |  |  |  |  |  |  |
| Typical angina | | 87 (24.9%) | 16 (19.5%) | 65 (34.4%) | 9 (15.3%) | 50 (22.8%) |
| Atypical angina | | 213 (60.9%) | 52 (63.4%) | 101 (53.4%) | 29 (49.2%) | 144 (65.8%) |
| Nonanginal chest pain | | 50 (14.3%) | 14 (17.1%) | 23 (12.2%) | 21 (35.6%) | 25 (11.4%) |
| **LVEF >= 50%** | | 334 (95.4%) | 78 (95.1%) | 181 (95.8%) | 59 (100%) | 204 (93.2%) |
| **Pre-test probability of CAD** | | 49 [33-59] | 47 [28-58] | 49 [28-69] | 34 [20-49] | 49 [37-59] |
| **Obstructive CAD** | | 98 (28.0%) | 26 (31.7%) | 50 (26.5%) | 15 (25.4%) | 69 (31.5%) |

**^#^** P value < 0.05 for comparisons among groups

CAD = coronary artery disease; CTCA = computed-tomography-coronary-angiography; CMR = cardiac-magnetic-resonance; ECHO = stress-echocardiography; LVEF = left-ventricular-ejection-fraction; PET = positron-emission-tomography; SPECT = single-photon-emission-computed-tomography.

**Table S2. Diagnostic performance of the different imaging strategies.**

|  | TP | TN | FP | FN | Uncertain |
| --- | --- | --- | --- | --- | --- |
| No-Imaging | - | 252 (72.0%) | - | 98 (28.0%) | - |
| CMR | 14 (17.1%) | 51 (62.2%) | - | 11 (13.4%) | 6 (7.3%) |
| ECHO | 10 (5.3%) | 70 (37.0%) | 4 (2.1%) | 16 (8.5%) | 89 (47.1%) |
| PET | 10 (16.9%) | 29 (49.2%) | 8 (13.6%) | 2 (3.4%) | 10 (16.9%) |
| SPECT | 38 (17.4%) | 99 (45.2%) | 14 (6.4%) | 20 (9.1%) | 48 (21.9%) |
| CTCA | 55 (15.7%) | 172 (49.1%) | 19 (5.4%) | 16 (4.6%) | 88 (25.1%) |
| CTCA-CMR | 15 (18.3%) | 47 (57.3%) | 7 (8.5%) | 10 (12.2%) | 3 (3.7%) |
| CTCA-ECHO | 35 (18.5%) | 118 (62.4%) | 9 (4.8%) | 8 (4.2%) | 19 (10.1%) |
| CTCA-PET | 11 (18.6%) | 39 (66.1%) | 4 (6.8%) | 4 (6.8%) | 1 (1.7%) |
| CTCA-SPECT | 48 (21.9%) | 125 (57.1%) | 14 (6.4%) | 19 (8.7%) | 13 (5.9%) |
| CMR-CTCA | 14 (17.1%) | 54 (65.9%) | - | 11 (13.4%) | 3 (3.7%) |
| ECHO-CTCA | 26 (13.8%) | 118 (62.4%) | 9 (4.8%) | 17 (9.0%) | 19 (10.1%) |
| PET-CTCA | 11 (18.6%) | 34 (57.6%) | 9 (15.3%) | 4 (6.8%) | 1 (1.7%) |
| SPECT-CTCA | 44 (20.1%) | 122 (55.7%) | 17 (7.8%) | 23 (10.5%) | 13 (5.9%) |
| ICA | 98 (28.0%) | 252 (72.0%) | - | - | - |

CMR = cardiac-magnetic-resonance; CTCA = computed-tomography-coronary-angiography; ECHO = stress-echocardiography; ICA= invasive-coronary-angiography; ICER = incremental-cost-effectiveness-ratio; PET = positron-emission-tomography; SPECT = single-photon-emission-computed-tomography

TN= true-negatives; TP= true-positives.

**Table S3. Centre specific reimbursement value for the different tests, values are expressed in local currency.**

|  | ICA | CTCA | CMR | ECHO | PET | SPECT |
| --- | --- | --- | --- | --- | --- | --- |
| Pisa, Italy | 2,512 € | 204 € | 310 € | 60 € | 1,072 € | 270 € |
| Viareggio, Italy | 2,512 € | 204 € | - | 60 € | - | 270 € |
| Naples, Italy | 1,737 € | 169 € | 310 € | 50 € | - | 270 € |
| Genoa, Italy | 1,737 € | 158 € | - | 62 € | - | 270 € |
| Firenze, Italy | 2,512 € | 204 € | - | 60 € | 1,072 € | 270 € |
| London, United Kingdom | 1,052 £ | 108 £ | 207 £ | - | - | 406 £ |
| Barcelona, Spain | 1,980 € | 197 € | 276 € | 156 € | - | 260 € |
| Madrid, Spain | 1,237 € | 279 € | - | 191 € | - | 260 € |
| Paris, France | 1,834 € | 141 € | 272 € | 165 € | 970 € | 450 € |
| Warsaw, Poland | 1,632 zl | 495 zl | - | 370 zl | - | - |
| Göppingen, Germany | 1,207 € | 509 € | 400 € | 82 € | - | - |
| Zurich, Switzerland | 3,919 Fr | 784 Fr | 539Fr | - | 1,253 Fr | 1,616 Fr |
| Turku, Finland | 1,146 € | 491 € | 656 € | 100 € | 945 € | - |

CMR = cardiac-magnetic-resonance; CTCA = computed-tomography-coronary-angiography; ECHO = stress-echocardiography; ICA= invasive-coronary-angiography; PET = positron-emission-tomography; SPECT = single-photon-emission-computed-tomography
